# High Temperature Cycles Result in Maternal Transmission and Dengue Infection Differences Between *Wolbachia* Strains in Aedes aegypti

**DOI:** 10.1128/mBio.00250-21

**Published:** 2021-11-09

**Authors:** Maria Vittoria Mancini, Thomas H. Ant, Christie S. Herd, Julien Martinez, Shivan M. Murdochy, Daniel D. Gingell, Enock Mararo, Paul C. D. Johnson, Steven P. Sinkins

**Affiliations:** a MRC-University of Glasgow Centre for Virus Research, Glasgow, UK; b Institute of Biodiversity, Animal Health and Comparative Medicine, University of Glasgow, Glasgow, UK; EPFL

**Keywords:** *Aedes*, *Wolbachia*, dengue, mosquito

## Abstract

Environmental factors play a crucial role in the population dynamics of arthropod endosymbionts, and therefore in the deployment of *Wolbachia* symbionts for the control of dengue arboviruses. The potential of *Wolbachia* to invade, persist, and block virus transmission depends in part on its intracellular density. Several recent studies have highlighted the importance of larval rearing temperature in modulating *Wolbachia* densities in adults, suggesting that elevated temperatures can severely impact some strains, while having little effect on others. The effect of a replicated tropical heat cycle on *Wolbachia* density and levels of virus blocking was assessed using Aedes aegypti lines carrying strains *w*Mel and *w*AlbB, two *Wolbachia* strains currently used for dengue control. Impacts on intracellular density, maternal transmission fidelity, and dengue inhibition capacity were observed for *w*Mel. In contrast, *w*AlbB-carrying *Ae. aegypti* maintained a relatively constant intracellular density at high temperatures and conserved its capacity to inhibit dengue. Following larval heat treatment, *w*Mel showed a degree of density recovery in aging adults, although this was compromised by elevated air temperatures.

## INTRODUCTION

*Wolbachia* are maternally inherited bacterial endosymbionts that naturally infect many arthropod species. The spread through host populations occurs by increasing the relative fitness of carriers in various ways, including reproductive manipulations such as cytoplasmic incompatibility (CI) (1). CI occurs when a *Wolbachia*-carrying male mates with a *Wolbachia*-free female, and results in reduced egg hatching. The major arbovirus mosquito vector Aedes aegypti is not a native *Wolbachia* host (1); however, artificial transfers have been carried out in the laboratory with a range of *Wolbachia* strains, some of which induce strong CI and greatly reduce the competence of *Ae. aegypti* to transmit arboviruses, including dengue and Zika (2–7). A new dengue control strategy utilizes CI to spread *Wolbachia* through wild mosquito populations and maintain it at high frequency, thereby reducing virus transmission. An increasing number of dengue endemic countries are incorporating releases of *Wolbachia*-carrying *Ae. aegypti* as part of ongoing dengue control efforts. Open-field release programs are under way in Indonesia, Vietnam, Australia, Malaysia, Colombia, and Brazil, with significant reductions in dengue incidence reported (8–10). Several *Wolbachia* strains have been stably introduced into *Ae. aegypti*, with different strains generating distinct fitness and pathogen blocking profiles. In particular, the *Wolbachia* strains *w*AlbB and *w*Mel, native to Aedes albopictus and Drosophila melanogaster, respectively, displayed the most promising characteristics in laboratory studies (2, 5, 7, 11, 12) and are both currently being deployed for dengue control.

*w*Mel belongs to the supergroup A *Wolbachia* clade. It provides protection from RNA viruses in its native host (13, 14), and blocks the transmission of dengue (DENV), chikungunya (CHIKV), and Zika (ZIKV) viruses in *Ae. aegypti* (2, 6, 15). The *w*Mel infection has been successfully established in *Ae. aegypti* populations in the cities of Cairns and Townsville in northern Australia, and in Yogyakarta, Indonesia, with data indicating reductions in cases of locally acquired dengue (9, 10, 16–18). *w*AlbB belongs to the supergroup B *Wolbachia* clade, and also efficiently blocks DENV and ZIKV transmission in *Ae. aegypti* (3, 7, 19). Open-field releases of *w*AlbB in Kuala Lumpur, Malaysia, have resulted in high population frequencies and significant reductions in dengue incidence (8, 20).

The magnitude of *Wolbachia*-mediated virus blocking usually shows a positive correlation with intracellular density (21, 22). Fitness costs also correlate with density, and high density strains cause both higher fitness costs and strong viral inhibition (2, 7, 23–25), although there are some exceptions (7, 26). *w*Mel and *w*AlbB reach comparable densities in female *Ae. aegypti*, and under standard laboratory conditions they show approximately equivalent levels of dengue (7, 11) and Zika blocking (7), and both have minimal effects on host fitness (2, 7, 27).

Invasiveness and stability of a *Wolbachia* strain depends primarily on CI induction capacity, maternal transmission efficiency, and effects on host fitness. The likelihood that a female will mate with a *Wolbachia*-carrying male and incur the fitness cost of CI increases with *Wolbachia* frequency. The fitness advantage of CI is therefore frequency dependent, with invasiveness following bi-stable dynamics determined by an invasion threshold (28, 29). Above the threshold the fitness advantages of CI overcome other fitness costs and *Wolbachia* will tend to spread; below the threshold fitness costs dominate and *Wolbachia* will tend to be lost. The high density *w*MelPop strain induces strong CI, but results in fitness costs over a range of life history traits, including reductions in longevity and the survival of eggs following periods of desiccated quiescence. *w*MelPop carrying *Ae. aegypti* were released in field sites in Australia and Vietnam and, despite reaching high initial infection frequencies, the strain was eventually lost once releases ceased (30).

Exposure of host insects to thermal stress is known to unbalance and perturb long-term symbiotic interactions and their phenotypes (31, 32), and *Wolbachia* frequency in insect populations can fluctuate seasonally and between geographical locations (33–35). In mosquitoes, several recent studies have demonstrated an impact of larval rearing temperatures on *Wolbachia* density in the resulting adults, with results suggesting that elevated temperatures can significantly reduce the density of some strains (7, 36, 37). *w*Mel appears to be particularly sensitive to high temperatures, with density dropping by several orders of magnitude when larvae are exposed to diurnal heat cycling between 27°C and 37°C. In these experiments, a reduced capacity of male carriers to induce CI and a lower level of maternal transmission were observed, with eventual loss of the strain when high rearing temperatures were maintained for more than one generation (36). In contrast, *w*AlbB was found to be more stable at high temperatures, with little (7) or no (36) reduction in density.

Previous studies have investigated the effects of high larval rearing temperatures on *Wolbachia* density in whole mosquitoes, and have examined effects on the transmission fidelity (7, 36, 37). However, reduced densities also suggest the potential for reduced virus blocking. Here, we examine the effects of simulated tropical temperatures on *Wolbachia* parameters and dengue transmission in transinfected lines of *Ae. aegypti*.

## RESULTS

### Effects of field-simulated temperature cycles on *Wolbachia* density.

**FIG 1:**
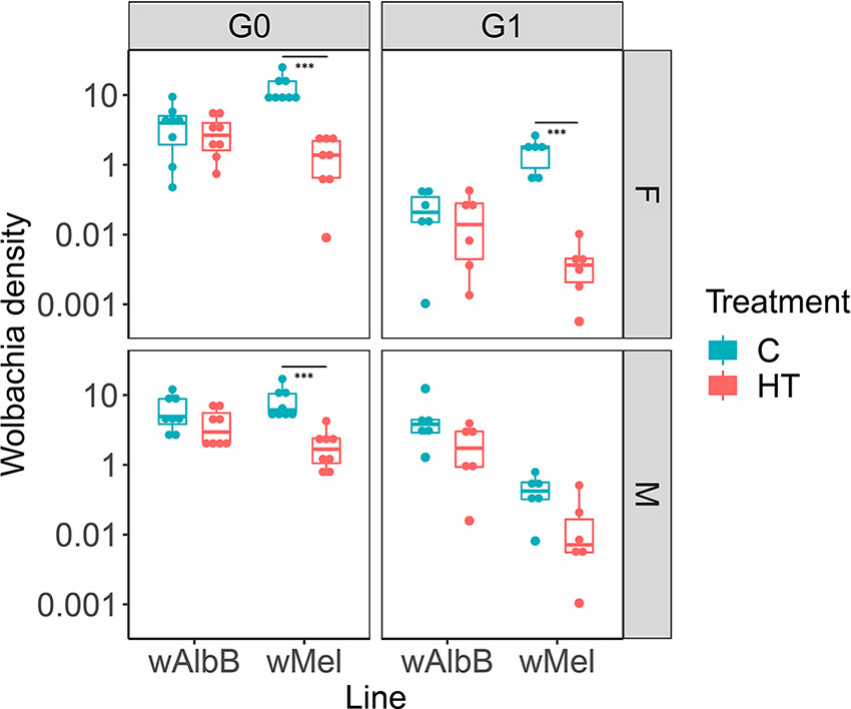
*Wolbachia* density in whole bodies of control (C, constant 27°C) and heat-treated (HT, temperature min = 28°C; temperature max = 36°C) mosquitoes. The densities of *w*AlbB and *w*Mel were quantified by qPCR on 5-day-old females (F) and males (M) over two generations of heat-treatment. Boxplots represent six biological replicates. Central line indicates the median of densities and whiskers represent upper and lower extremes. T-test and Mann-Whitney test were used for statistical analyses. *** 0.001.

Detailed temperature recordings from tropical *Ae. aegypti* larval breeding sites were obtained from a previously published study (38), and a replica-cycle (temperature min: 28°C; max: 36°C, Fig. S1) was generated in the laboratory using a programmable dynamic-temperature cabinet (henceforth “heat treatment”). Larvae from *w*Mel- and *w*AlbB-carrying *Ae. aegypti* lines were reared under heat or control (constant 27°C) conditions. On eclosion, adult mosquitoes from both treatments were maintained at a constant 27°C. Five-day-old adults were sacrificed, and *Wolbachia* densities assessed (Fig. 1). Subsets of females were blood-fed, and the resulting progeny exposed to a second round of larval heat treatment, with *Wolbachia* densities in G1 adults assessed 5 days after emergence. Consistent with previous studies (7, 36, 37), the *w*Mel strain was particularly susceptible to density reductions resulting from heat treatment, with a significant drop in density in females from 12.6 ± 5.9 *Ct wsp/Ct HTH* (mean ± SD) to 1.4 ± 0.9 *Ct wsp/Ct HTH* (*P  < * 0.001, Mann-Whitney test) after one generation of heat treatment. These results confirm that the density drop did not result from a sudden heat shock resulting from the two-temperature diurnal regime used in previous studies. A similar trend was observed in female adults resulting from a second larval heat treatment, decreasing from 1.5 ± 0.7 *Ct wsp/Ct HTH* to 0.04 ± 0.03 *Ct wsp/Ct HTH* (*P  < * 0.001, unpaired *t* test). *w*Mel density decreased also in male adults, significantly dropping after one round of larval heat treatment (*P  <* 0.001, Mann-Whitney test); in contrast, the second larval heat treatment on the male progeny had a milder effect, causing a slight decrease (not significant, *P  = * 0.06) in *w*Mel density. The *w*AlbB strain maintained a constant density over both generations of heat treatment in females and males (*p* > 0.5 for both generations and sexes, Unpaired *t* test and Mann-Whitney test).

10.1128/mBio.00250-21.1 FIG S1 Simulated larval, adult regime 1, and adult regime 2. (A) A representative 24-h period of the simulated tropical water-temperature cycle generated from data from water drums known to act as *Ae. aegypti* larvae breeding sites in Trinidad (38). Data was collected using a waterproof temperature probe placed in a volume of water equal to that of the larval pans, and left in a dynamic temperature incubator. 24-h period for regime 1 (B) and 2 (C**)** cycles for adult temperatures generated from data collected in urban Kuala Lumpur. Readings are from a temperature probe placed in a dynamic temperature incubator running the replica cycle. Download FIG S1, PDF file, 0.2 MB. Copyright © 2021 Mancini et al. 2021 Mancini et al. https://creativecommons.org/licenses/by/4.0/ This content is distributed under the terms of the Creative Commons Attribution 4.0 International license.

### Effects of field-simulated temperature cycles on *Wolbachia* maternal transmission.

**FIG 2:**
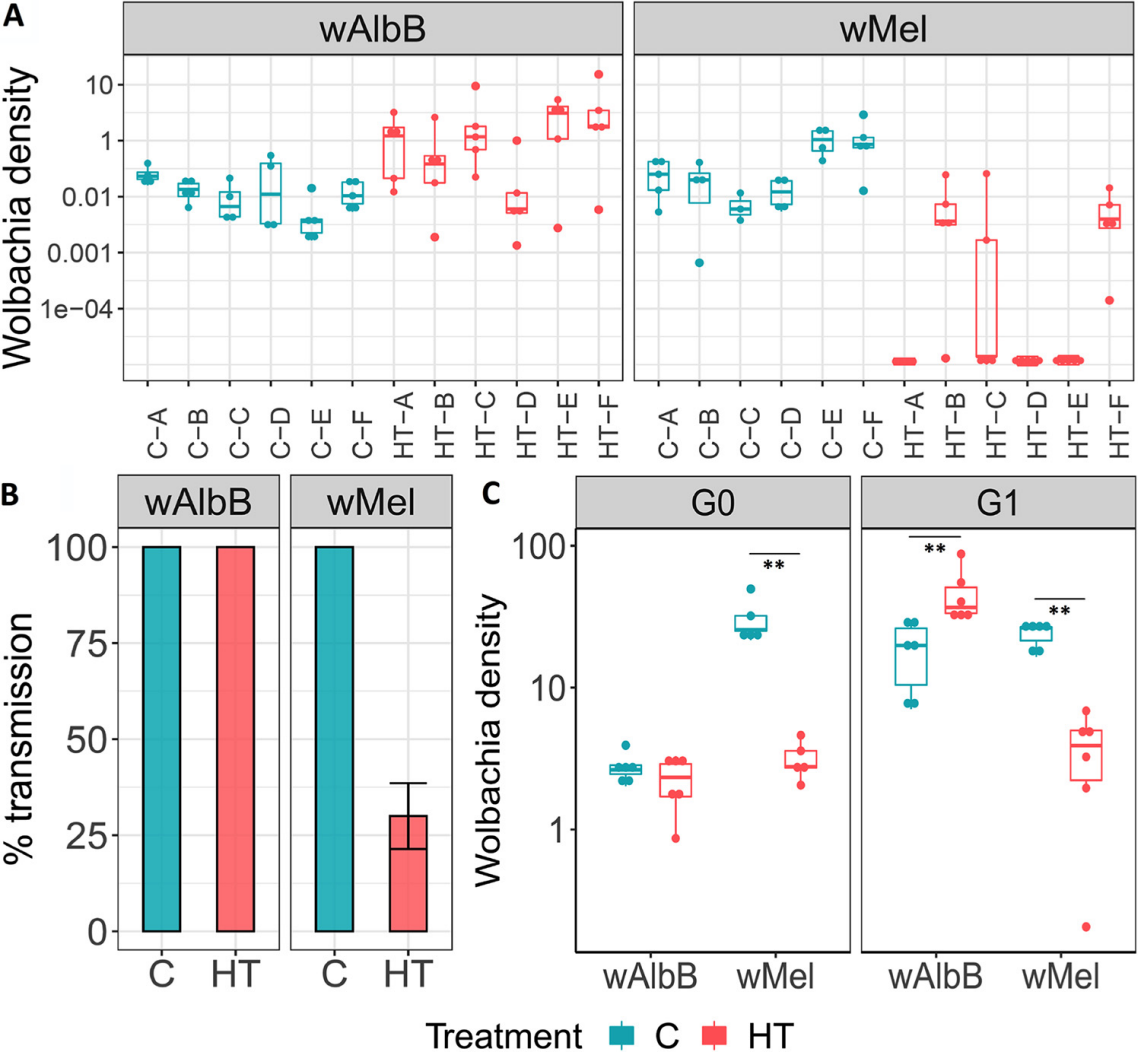
Whole-body densities, maternal transmission rate, and ovary-specific densities of *w*AlbB and *w*Mel in control (C, constant 27°C) and heat-treated (HT, temperature min  =  28°C; temperature max 36°C) mosquitoes. (A) Progeny from single females reared as larvae under C or HT conditions were hatched in families (A, B, C, D, E, F) and reared at 27°C. Six L4 larvae were randomly sampled from each individualized female and assessed for *Wolbachia* density by qPCR (A) and infection-status by strain-specific PCR (*N*  =  60 for each treatment/strain). (B). *Wolbachia* density data were computed using linear mixed models with fixed (*Wolbachia* line, treatment) and random effect (families), followed by multiple comparisons and *P values* adjustment. (C) Densities of *w*AlbB and *w*Mel were measured in six pools of three sets of dissected ovaries. The center of a box indicates the median of densities and whiskers represent upper and lower extremes. A Mann-Whitney test was used. ** 0.01; * 0.05.

*Wolbachia*-carrying females reared under either heat treatment or control conditions were back-crossed to wild-type males. Females were individualized for oviposition and the resulting G1 eggs hatched as single families. G1 larvae were reared at a constant 27°C until the L4 stage, whereupon a random selection from each family was assessed for *Wolbachia* status and density. Comparisons between models showed a significant effect of the *Wolbachia* strain (*P*  <  0.001, χ^2^_df=1_ = 18.2) and of the interaction between the strain of *Wolbachia* and the high-temperature larval treatment (*P*  <  0.001 χ^2^_df=3_ = 67.6). *w*Mel densities in the *Wolbachia* positive G1 progeny were significantly lower following heat treatment than densities in the G1 progeny following control treatment (*p* < 0.001) (Fig. 2A); endpoint PCR showed that the maternal transmission of *w*Mel was significantly reduced following larval heat treatment, with the complete loss of *w*Mel in three of the six heat-treated families, compared with 100% transmission in the control group (*P*  <  0.001, Fisher’s exact test) (Fig. 2B).

In contrast, *w*AlbB females resulting from larvae reared under either heat treatment or control conditions transmitted *Wolbachia* to 100% of offspring. Interestingly, the G1 progeny from heat treated *w*AlbB mothers displayed higher *Wolbachia* densities compared to progeny resulting from mothers reared under control conditions (*P* <  0.001). A strong significant difference in *Wolbachia* load was also observed between the progeny of *w*AlbB and *w*Mel heat-treated females (*P*  <  0.001) (Fig. 2A).

To associate perturbations in maternal transmission with *Wolbachia* densities in ovaries, females reared under either heat treatment or control conditions were dissected, and ovary densities assessed by qPCR. Results indicate that heat treatment caused significant reductions in the ovary density of *w*Mel, while the density of *w*AlbB was not negatively affected, and even increased (*P*  =  0.002, Mann-Whitney test), compared with controls following two generations of treatment (Fig. 2C). Effects on *Wolbachia* density in mosquito ovaries can also be visualized by whole-mount fluorescent *in situ* hybridization (FISH) (Fig. S2).

10.1128/mBio.00250-21.2 FIG S2 Fluorescent *in situ* hybridization. Visualization of distributions and density reductions of *Wolbachia* (green) in ovaries of 5-day-old females from *w*Mel, *w*AlbB, and wild-type *Ae. aegypti* females from control and heat-treated groups. Blue stain is DAPI. Download FIG S2, PDF file, 1.0 MB. Copyright © 2021 Mancini et al. 2021 Mancini et al. https://creativecommons.org/licenses/by/4.0/ This content is distributed under the terms of the Creative Commons Attribution 4.0 International license.

### Effects of field-simulated temperature cycles on virus transmission.

**FIG 3:**
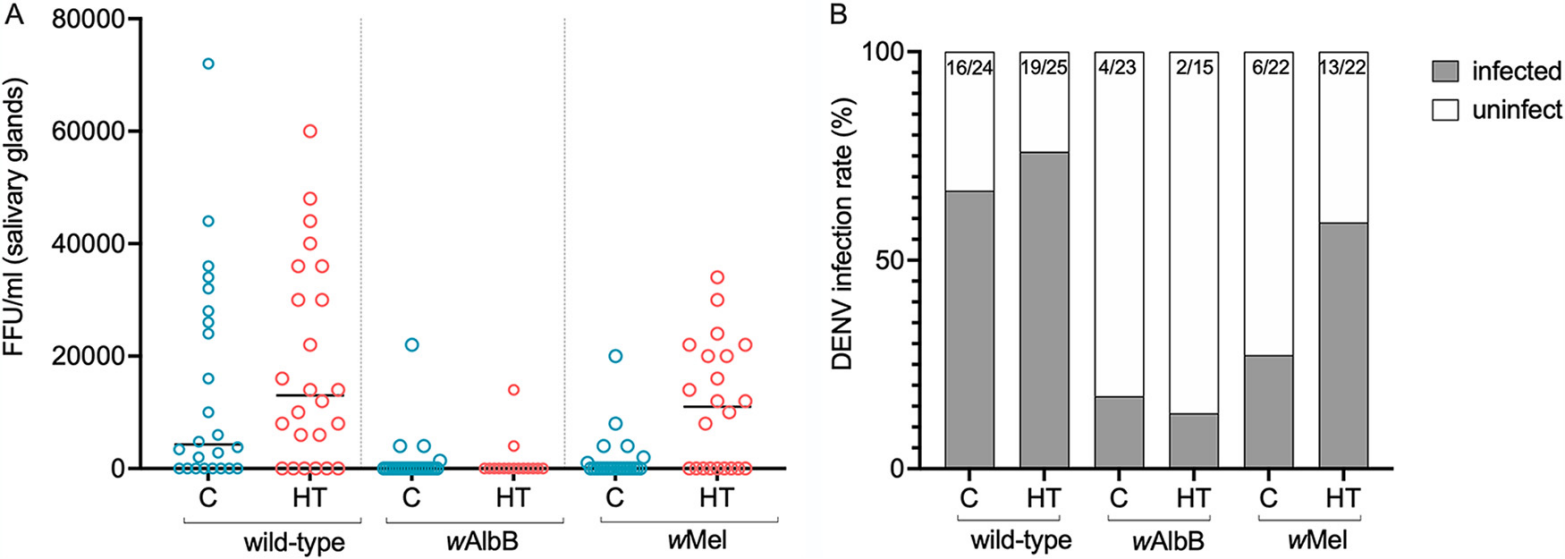
Effect of larval heat-treatment on dengue inhibition. Wild-type, *w*AlbB-, and *w*Mel-carrying females were fed on DENV2-infected blood-meal. Engorged females were selected and incubated for 12 days. Salivary glands from control and heat-treated females were assessed for virus dissemination by FFA. Dots represent the number of foci/ml and each dot corresponds to a single mosquito (A). Dissemination rates from the same experiments are represented in panel B. Proportions on each bar indicate the infection rates (*N* positive/*N* total). Statistical analysis was performed using a generalized linear model.

To test whether temperature-induced changes in the *Wolbachia* lines (assumed to be associated with *Wolbachia* density changes) could impact dengue inhibition, larvae from the *w*AlbB, *w*Mel and wild-type lines were reared under either heat treatment or control conditions, and the resulting 5-day old adult females were orally challenged with a bloodmeal containing DENV-2. Twelve days postfeeding, levels of infectious virus in mosquito tissues were quantified to assess infection and transmission potential within the vector (Fig. 3, Fig. S3).

10.1128/mBio.00250-21.3 FIG S3 DENV challenge on heat-treated wild-type, *w*AlbB-, and *w*Mel-carrying *Ae. aegypti.* Salivary glands from heat-treated females were assessed for virus dissemination by fluorescent focus assay (FFA). Dots represent the number of foci/ml per single mosquito (A). Dissemination rates from the same experiments are represented in panel B. Proportions on each bar indicate the infection rates (*N* positive/*N* total). Statistical analysis was performed using Mann-Whitney test and Fisher’s exact test. *** 0.001; ** 0.01; * 0.05; ns, not significant. Download FIG S3, PDF file, 0.2 MB. Copyright © 2021 Mancini et al. 2021 Mancini et al. https://creativecommons.org/licenses/by/4.0/ This content is distributed under the terms of the Creative Commons Attribution 4.0 International license.

We tested the effects of strain, heat treatment, and their interaction on both the probability of viral infection and the intensity of infection (viral load) given that infection had occurred. The ability of *w*Mel to affect viral infection intensity was dependent on whether heat treatment or control larval rearing was used (*P*  =  0.048, χ^2^_df=1_ = 3.92). *w*Mel mosquitoes displayed significantly lower viral infection titer compared with wild-type when reared at standard control conditions (*P*  =  0.005, χ^2^_df=1_ = 7.98), but there was a difference between the strains when reared under heat treatment (*P*  =  0.365, χ^2^_df=1_ = 0.82) (Fig. 3A). In addition, *w*Mel mosquitoes showed a higher probability of infection inhibition than wild type irrespective of larval rearing temperature (*P*  =  0.006, χ^2^_df=1_ = 7.59)—only six mosquitoes out of 22 were found positive for DENV viral particles (27%) (Fig. 3A and B). There was also an increased probability of infection in the heat treatment cohort regardless of strain (*P*  =  0.047, χ^2^_df=1_ = 3.95): 59% (13/22) of heat-treated mosquitoes were found to be infected (Fig. 3B).

*w*AlbB maintained strong viral inhibition following high-temperature rearing, showing a highly significant effect of the strain on viral infection intensity and prevalence, compared with the wild-type group (Fig. 3A and B). *w*AlbB had a negative effect on viral load in salivary glands (*P*  =  0.020, χ^2^_df=1_ = 5.42) regardless of larval rearing regime, and significantly decreased the probability of infection (*P*  <  0.001, χ^2^_df=1_ = 27.70), with the odds ratio for infection relative to the wild-type control of 0.077.

From an independent viral challenge, a similar pattern of infection was observed in the salivary glands of heat-treated mosquitoes (Fig. S3). *w*Mel females displayed a significant increase in viral titer (Fig. S3 A) and in infection rate (Fig. S3 B), compared with heat-treated *w*AlbB (*P*  <  0.001 Mann-Whitney test; *P*  <  0.001, Fisher’s exact test, respectively). While viral titers in salivary gland tissue were significantly lower in high-temperature-reared *w*AlbB females compared to wild-type females (*P  = * 0.006 Mann-Whitney test), no significant difference was observed between heat-treated *w*Mel and wild-type females (*P  =  *0.275, Mann-Whitney test).

### Adult exposure to elevated temperatures and Wolbachia recovery.

**FIG 4:**
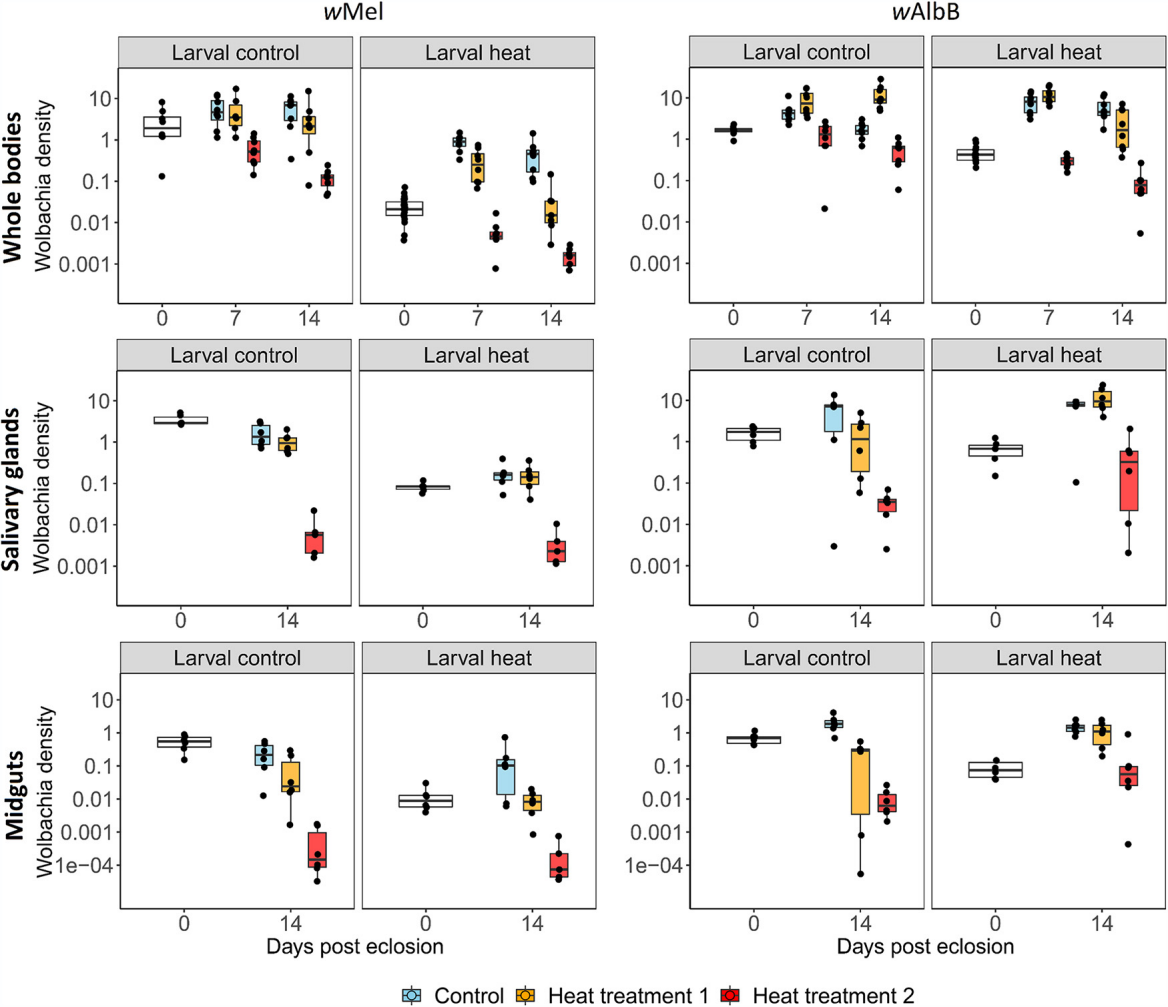
Effects of high temperature larval and adult ambient air temperatures on *w*Mel and *w*AlbB densities in whole-bodies, salivary-gland, and midgut tissues. Larvae were reared under control (larval control, constant 27°C) and high temperature (larval heat, temperature min = 27°C; temperature max 37°C) conditions. A subset of females was sampled immediately on eclosion (day 0), and the densities assessed. The remaining females were divided into three adult treatment temperatures: control (constant 27°C), adult regime 1 (temperature min  =  28°C; temperature max  =  33.5°C), and adult regime 2 (temperature min  =  27°C; temperature max  =  36.5°C). Adults were sampled and densities assessed in whole bodies (days 7 and 14 posteclosion) and dissected salivary gland and midgut tissues (day 14 posteclosion). Data points represent single whole adult females, or pools of three salivary glands or midguts.

A previous study reported substantial recovery of *w*Mel in adult *Ae. aegypti* from initially low densities following larval rearing at high temperatures (37). To further investigate density recovery in adults, and to examine the impact of elevated air temperatures on recovery rates, *w*Mel and *w*AlbB larvae were reared under control or high temperature conditions, with emerging adults exposed to replica heat cycles generated from recordings of ambient temperatures in shaded (henceforth “adult temperature regime 1”—temperature min: 28°C; max: 33.5°C) or semi-shaded (henceforth “adult temperature regime 2”—temperature min: 27°C; max: 36.5°C) sites in urban Kuala Lumpur (Fig. S1). Adult females were dissected, and *Wolbachia* densities in midgut and salivary gland tissues were assessed (Fig. 4).

There was a significant reduction in the density of *w*Mel in adults emerging from heat treatment (0.02 ± 0.01 *Ct wsp/Ct HTH*, mean ± SD) compared with control treatments (2.8 ± 2.6 *Ct wsp/Ct HTH*) (*p* < 0.001, Mann-Whitney test). However, there was a marked recovery in density in heat treated larvae subsequently reared under control conditions as adults (reaching 0.4 ± 0.4 *Ct wsp/Ct HTH* after 14 days), although this recovery was incomplete, with adults from control larvae maintaining a significantly higher density, 5.9 ± 3.7 *Ct wsp/Ct HTH* after 14 days (*p* = 0.003, Mann-Whitney test). Air temperature had a significant impact on the recovery of *w*Mel, with 14-day-old females from the adult regimes 1 and 2 displaying significantly lower densities than adults reared at control temperatures (*p* < 0.001 for both adult temperature regimes, Mann-Whitney test).

A similar trend was observed in dissected salivary gland and midgut tissues of emerging adults, with significant reductions in *w*Mel density in both tissues following larval heat treatment (*p* < 0.005 for both midguts and salivary glands, Mann-Whitney test). There was a recovery in density in midguts at day 14 in the heat treatment cohort reared at the control adult temperature, with no significant difference compared to mosquitoes reared exclusively under control conditions (*P = *0.48, Mann-Whitney test). Densities in the salivary glands of females reared under heat treatment showed minimal recovery at both control and adult temperature regime 1, with significantly lower densities compared with mosquitoes reared exclusively under control conditions (*p* < 0.005 for both control and adult temperature regime 1, Mann-Whitney test). Adults maintained under temperature regime 2 showed significant reductions in density in both salivary gland and midgut tissues compared with adults reared at control temperatures (*p* < 0.005 for both salivary glands and midguts, Mann-Whitney test).

*w*AlbB showed a reduction in density in adults emerging from heat treatment (0.4 ± 0.2 *Ct wsp/Ct HTH*) compared with control treatments (1.6 ± 0.4 *Ct wsp/Ct HTH*) (*p* <0.001, Mann-Whitney test). However, the density recovered fully after 7 days of adult rearing under control conditions, with no significant reduction in density compared to mosquitoes reared only at control temperatures (*P  = * 0.129, Mann-Whitney test). Interestingly, *w*AlbB-carriers reared at the control temperature as larvae and the adult temperature cycle 1 showed significantly increased *Wolbachia* densities compared with adults reared only under control conditions (*P  =  *0.002, Mann-Whitney test). Adult treatment 2 resulted in significant reductions in densities compared with adults reared at the control temperature, regardless of larval treatment (*p* < 0.001 for both control and heat-treated, Mann-Whitney test).

Following larval heat treatment, the density of *w*AlbB in both midgut and salivary gland tissues of eclosing adults was slightly but significantly reduced (*P  =  *0.02 for salivary glands; *P  = * 0.002 for midguts). However, densities in both tissues recovered fully at day 14 when adults were reared under either control or adult temperature regime 1 temperature conditions, displaying no significant reductions compared to tissue densities in adults reared under control conditions only (*p* > 0.18 for all comparisons). Similar to *w*Mel, rearing *w*AlbB adults under the temperature regime 2 resulted in significant reductions in densities compared to adults reared under control conditions, regardless of larval treatment.

## DISCUSSION

*Ae. aegypti* larvae developing in the tropics encounter a far broader and variable range of temperatures than those typically used in mosquito insectaries (usually stringently maintained in the range of 27°C –28°C). Several recent studies have highlighted the substantial influence that larval water temperature has in determining the density of some *Wolbachia* strains in *Ae. aegypti*, particularly *w*Mel (36, 37, 39). This is noteworthy as *Wolbachia* strain characterization is routinely performed under standard insectary temperatures and suggests that phenotypes predicted by laboratory tests may vary in the field. Tropical breeding sites can experience heating above 30°C for extended periods of the day, and in some cases reach daily maxima in excess of 36°C (38, 40). The high temperature regime used in this study was generated from data collected from water drums in Trinidad acting as *Ae. aegypti* larval habitats (38).

Consistent with previous studies, *w*Mel was found to be negatively affected by exposure to the high temperature cycle, showing a significant decrease in whole body density. A substantial drop in adult ovary density was also observed, leading to a reduction in maternal transmission of approximately 75%. Imperfect maternal transmission can impact the population stability of a *Wolbachia* infection by increasing the invasion threshold, potentially compromising the ability of *w*Mel to spread and persist in wild populations. Previous evidence documented a disruption in *w*Mel maternal transmission and CI induction following exposure to high temperatures (7, 36). Additionally, intense artificial laboratory selection for a heat resistant *w*Mel variant in *Ae. aegypti* failed to produce a strain with improved thermal tolerance, an observation that was supported by experiments showing that field collected *w*Mel-carriers from a hot climate did not differ substantially in their response to heat stress compared with a laboratory colony—suggesting that adaption of the strain to high temperatures may be intrinsically difficult (39). In contrast, the *w*AlbB strain showed relative heat stability when larvae were reared under the high temperature regime. High densities were maintained in the ovaries, resulting in complete maternal transmission, suggesting that the *w*AlbB strain would be more stable among populations in hot tropical climates.

For the first time, the consequences of tropical heat stress on the ability of *Wolbachia* to inhibit dengue virus dissemination was tested in *w*Mel and *w*AlbB-carrying *Ae. aegypti*. Rates of mosquito infection following challenge with DENV2 were quantified in order to predict the infective state of mosquitoes reared under either heat treatment or control conditions. Following exposure to the thermal stress of heat treatment conditions, *w*AlbB retained its ability to efficiently block DENV2 dissemination, while *w*Mel showed a significant increase in viral dissemination. *Wolbachia*-mediated viral inhibition is thought to be primarily cell autonomous (5, 41); consequently, densities in midgut and salivary gland tissues are key to blocking virus dissemination and transmission. The reduction in dengue inhibition in heat-treated *w*Mel is concomitant with large reductions in *Wolbachia* density in both midgut and salivary gland tissues, although the density in midguts appeared to recover in adults after 14 days. *w*AlbB also showed reductions in density in midgut and salivary gland tissues, although the reduction was not as dramatic as *w*Mel, and recovered fully in 14-day-old adults.

A decrease in the efficiency of dengue blocking by *w*Mel could have significant impacts on the utility of the strain as a vector control intervention in hot tropical climates. This is particularly relevant given the role of high temperatures as a covariate of dengue transmission (42). Moreover, there is the potential that a weakening of the *w*Mel transmission blocking phenotype following exposure to high temperatures could increase the risk of selection of virus escape mutations that confer a lower general susceptibility to *Wolbachia*-mediated inhibition—and could therefore undermine *Wolbachia* interventions. It should be noted that *Wolbachia* at high density induce a broad range of perturbations in *Ae. aegypti* cells (43), including in a number of pathways that are important in the flavivirus life cycle—such as lipid transport and metabolism, autophagy, vesicular trafficking, and endoplasmic reticulum stress; this is inherently likely to reduce the risk of selection of virus escape mutations. However, at lower density the levels of perturbation are reduced (43).

A previous study has shown that initially low densities of *w*Mel following larval heat treatment can recover substantially in adults reared under normal insectary conditions (37). Results presented here are consistent with this finding, with *w*Mel showing considerable (although incomplete) density recovery when adults are reared at a constant 27°C. However, while adult mosquitoes are able to fly and seek cooler resting areas, ambient air temperatures are often very high in the tropics. Recordings in shaded and semishaded sites from urban Kuala Lumpur indicate that air temperatures can reach in excess of 34°C for several hours of the day. In larvae carrying *w*Mel reared using the high temperature cycle, and subsequently reared as adults using a replica shaded air-temperature cycle (defined as adult temperature regime 1), only a limited recovery in *Wolbachia* density occurred. In contrast, the density of *w*AlbB in whole mosquitoes reared as adults using the temperature cycle 1 were significantly higher than controls—suggesting that the temperature optimal for *w*AlbB replication may actually be higher than the 27°C used in standard rearing. Both *w*Mel and *w*AlbB densities in adults were substantially reduced by exposure to the temperature cycle 2, suggesting that *w*AlbB is not completely resistant to the effects of high temperatures, although this cycle represents a temperature regime that adult mosquitoes will be unlikely to encounter for extended periods. The *w*AlbB strain was capable of reaching and maintaining high frequencies and significantly reducing dengue transmission in the hot tropical climate of urban Kuala Lumpur, Malaysia (8).

Although the *w*Mel strain used in this study shows reduced densities in the laboratory using simulated field conditions, releases in Australia, Brazil, and Indonesia demonstrate that *w*Mel can stably invade wild *Ae. aegypti* populations (9, 10, 16, 44, 45) and maintain its ability to block dengue (17, 46). In direct comparisons, *w*Mel line produced slightly lower fitness costs than *w*AlbB (7), suggesting that it may be the more invasive of the two strains in cooler climates. Exposure of *w*Mel-carrying *Ae. aegypti* adults to a diurnal temperature cycle with a mean of 28°C and a fluctuating range of 8°C (±4°C) caused a decrease in bacterial density when compared to constant 25°C, but did not reduce the ability of *Wolbachia* to inhibit dengue transmission (47). Moreover, in some hotter equatorial locations *Ae. aegypti* can exploit underground larval habitats, such as wells and drains, which will be away from direct sunlight and cooler than ground level. Laboratory experiments have also proved that the effects of thermal stress on *Wolbachia* density are stage-specific (35, 37); in particular, exposure of early larval stage generates a significant and irreversible decrease in density, while the drop observed during exposure to later stages is rescued during adulthood. This suggests that the variations in temperature typical of the field will result in a more complex gradient of phenotypes, less clear-cut than those produced in the laboratory. The complex interactions between environmental temperatures and *Wolbachia* phenotypes has been recently investigated in natural *Wolbachia*-*Drosophila* associations, where the developmental temperature of the host modulated *Wolbachia*-induced antiviral effects, ranging from complete to no protection, affecting *Wolbachia* density (higher at 25°C compared with 18°C); in contrast, postinfection exposure to different temperatures impairs viral protection without affecting symbiont density (48).

Our data confirm that high tropical temperatures have a significant impact on the phenotypic stability of *Wolbachia* in *Ae. aegypti*, and the magnitude of this impact varies substantially between *Wolbachia* strains. Of the strains currently used in open field releases, *w*Mel appears to be particularly susceptible and *w*AlbB relatively stable under thermal stress, with *w*Mel displaying a marked reduction in capacity for maternal transmission and dengue blocking—which is not observed with *w*AlbB. The selection for the optimal strain for *Wolbachia*-deployed vector control strategies must therefore consider phenotypic stability in relation to the geography and climate of selected intervention areas. The water temperature of natural breeding sites not only represents a crucial abiotic factor known to directly affect vector biology (49), but it also plays a role in ensuring the most effective *Wolbachia*-based strategy for reducing dengue transmission.

## MATERIALS AND METHODS

### Mosquito rearing.

*w*Mel, *w*AlbB, and wild-type *Ae. aegypti* mosquitoes were derived from previously generated lines (7), sharing the same genetic background. Colonies were maintained at 27°C and 70% relative humidity with a 12-h light/dark cycle. Larvae were fed with tropical fish pellets (Tetramin, Tetra, Melle, Germany) and adults maintained with 5% sucrose solution *ad libitum*. Blood meals were provided using an artificial blood-feeding system (Hemotek, UK) using human blood (Scottish National Blood Transfusion Service, UK). Eggs were collected on a wet filter-paper (Grade 1 filter paper, Whatman plc, GE health care, UK). Eggs were desiccated for 5 days and later hatched in deionized water containing 1g/L bovine liver powder (MP Biomedicals, Santa Ana, CA, USA).

### Temperature cycles.

For each replicate performed over multiple generations, eggs from *w*Mel, *w*AlbB, and wild-type (WT) *Ae. aegypti* lines were hatched and separated into experimental groups: larval density (200 larvae per 500 ml of water) and food were consistent between the conditions.

Heat-challenged larvae were maintained in Panasonic MLR-352-H Plant Growth Chamber incubator (Panasonic, Osaka, Japan). The applied temperature regime was based on data from *Ae. aegypti* larval breeding containers in Trinidad (38) and replicated in the cabinets. Water temperatures were continuously monitored using a data logger (Hobo Water Temperature Pro V2, Bourne, MA, USA) placed in a plastic tray filled with 500 ml of water. Temperature data were registered and monitored. Mosquitoes under control conditions were stably maintained at 27°C, as described above. Pupae were sexed according to size, introduced into cages and maintained during the adult stage at 27°C, unless otherwise stated.

For assessing *Wolbachia* recovery during the adult stage, females from control and heat-treated groups were selected and divided into three different adult treatments: (i) C: control (27°C constant); (ii) adult temperature regime 1 (temperature peak at 32°C); and (iii) adult temperature regime 2 (temperature peak at 37°C). Temperature cycles for adult treatments are based on air temperature readings registered in Kuala Lumpur in February 2019. Readings for the adult temperature regime 1 were collected in the area of Pusat Komersial Shah Alam (3°03'57.2"N 101°29'24.0"E) in a shaded location, while a semishaded area of the Institute of Medical Research (3°10'10.3"N 101°41'55.0"E) was used for the adult regime 2.

### *Wolbachia* density and fluorescent in situ hybridization.

Genomic DNA from 5- to 7-day-old (unless otherwise stated) whole females and males of *Wolbachia*-carrying lines was extracted with STE buffer (10uM Tris HCL pH 8, 100 mm NaCl, 1 mm EDTA) and used for *Wolbachia* density quantification by qPCR using the relative quantification of the *Wolbachia* surface protein (*wsp*) gene against the homothorax gene (HTH) as reference gene. The following program was used to run the qPCRs: 95°C for 5 min, 40× cycles of 95°C for 15 s and 60°C for 30 s, followed by a melt-curve analysis. A Rotor Gene Q (Qiagen) was used with 2x QuantiNova SYBR.

Ovaries, salivary glands, and midguts (six pools of three organs per each replicate) were dissected from 5-day-old females using sterile forceps and needles in a drop of sterile PBS buffer, and immediately transferred into tubes containing STE buffer; genomic DNA from tissues was extracted and *Wolbachia* density was assessed by qPCR as previously described. Data shown in the plots are the representation of density quantitation of one of three independent biological replicates, consistently showing the same trend of results.

At the same time, ovaries were also dissected for FISH in sterile PBS buffer, and then immediately transferred to a tube containing Carnoy’s fixative (chloroform:ethanol:acetic acid, 6:3:1) and fixed at 4°C overnight. Samples were then rinsed in PBS and incubated in a hybridization buffer containing: 50% formamide, 25% 20 × SSC, 0.2% (wt/vol) dextran sulphate, 2.5% herring sperm DNA, 1% (wt/vol) tRNA, 0.015% (wt/vol) DTT, 1% Denhardt’s solution, and 100 ng/ml of each probe. The probes annealed on the *wsp* gene (5). Samples were left to hybridize overnight in a dark-humid box at 37°C. Samples were washed twice in a solution containing: 5% 20 × SSC, 0.015% (wt/vol) DTT, and twice in a solution of 2.5% SSC, 0.015% (wt/vol) DTT in dH2O, and incubated at 55°C for 20 min. Samples were then placed on a slide containing a drop of VECTASHIELD Antifade Mounting Medium with DAPI (Vector Laboratories, CA, USA) and were visualized immediately using a confocal microscope (ZEISS, Germany).

### *Wolbachia* recovery during adult stage.

After larval treatments, named control (Larval Control) and heat-treated (Larval Heat), eight females from different experimental groups denoted as larval-treatment/adult-treatment as follows: control/control; control/adult regime 1; control/adult regime 2; heat/control; heat/adult regime 1; heat/adult regime 2, were sampled after 0, 7, and 14 days. Midguts and salivary glands were also dissected a few hours after eclosion (day 0) and after 14 days. *Wolbachia* density was assessed in whole mosquitoes and tissues by qPCR as previously described.

### Maternal transmission.

Maternal transmission of each *Wolbachia* strain after heat stress was evaluated by backcrossing heat-treated females with heat-treated wild-type males, while control females mated with control wild-type males. After offering a blood-meal, 10 engorged females per group were selected and, after 3 days, individualized on damp circle of filter paper inside up-turned plastic cups. Filter papers were collected and individually desiccated. Once dried, eggs were hatched in containers and reared at stable control temperature; 6 to 10 fourth-instar larvae were randomly sampled from each individualized female (10 females) and assessed for *Wolbachia* infection by PCR, using strain specific primers described in Table S1. PCRs were set up using 1× Taqmaster mix (Vazyme) according to the manufacture’s protocol**;** the amplification reaction consisted of a cycle at 94°C for 3 min, followed by 30 cycles of denaturation at 94°C for 30 s, annealing at 55°C for 30 s, extension at 72°C for 30s, and a final step at 72°C for 10 min. Additionally, a subset of samples (six individuals for six family) were validated by qPCR, using *wsp* general primers.

10.1128/mBio.00250-21.4 TABLE S1 List of sequences of oligonucleotides and probes. Download Table S1, PDF file, 0.05 MB. Copyright © 2021 Mancini et al. 2021 Mancini et al. https://creativecommons.org/licenses/by/4.0/ This content is distributed under the terms of the Creative Commons Attribution 4.0 International license.

### Virus challenge.

Five-day-old females per group were fed an infectious blood-meal consisting of human blood and DENV serotype-2 virus (New Guinea C Strain). The virus was serially passaged in *Ae*. *albopictus* C6/36 cells: the infected supernatant was harvested, concentrated using Amicon Ultra-15 filters (Millipore, IRL), and titered *via* fluorescent focus assay (FFA), as described below. Two independent challenges were carried out using different batches of propagated virus at the final concentration in the blood of 1.9 × 10^7^ FFU/ml and 1.7 × 10^7^ FFU/ml, respectively. Fifty control and heat-treated females were infected for each challenge. Fully engorged females were transferred to a climatic chamber at 27°C, 70% relative humidity, and a 12-h light/dark cycle, and maintained on 5% sucrose solution. After 12 days, mosquitoes were dissected and sampled: for the first challenge, salivary glands of control and heat-treated females were used to quantify virus titers, while only heat-treated individuals were involved for the second analysis. Samples were transferred in Dulbecco’s Modified Eagle Medium (DMEM) supplemented with 2% fetal bovine serum (FBS). After homogenizing, 10-fold serial dilutions (10^−1^ to 10^−3^) of each solution was transferred onto a monolayer of Vero cells for viral quantification with FFA. Primary antibody for DENV was the MAB8705 anti-dengue virus complex antibody (Millipore); secondary antibody was the Goat anti-mouse Alexa Fluor 488, A-11001 (Thermo Scientific, Waltham, MA, USA). A Celigo Imaging Cytometer (Nexcelom Bioscience, Lawrence, MA, USA) was used for imaging plates. Fluorescent foci were counted by eye (from dilutions with less than 100 foci) and virus titers calculated and expressed as FFU/ml.

### Statistical analysis.

Graphics were generated using the *ggplot2* package (50) of the *R* software (version 3.6.1) (51) and Prism Software (version 9). Statistical tests were run using Prism version 9 and R. A Shapiro-Wilk test was used for assessing normality distribution of data, and parametric and nonparametric tests were selected accordingly for assessing *Wolbachia* density in mosquitoes. *Wolbachia* density data in the maternal transmission assay were analyzed with linear mixed models (*lme4* package) (52) after log_10_ transformation to meet the assumptions of normality. Post hoc pairwise multiple comparisons were performed with the function *emmeans* (R package emmeans) (53) and *P values* adjusted using Bonferroni method.

Viral titers and infection prevalence were analyzed as the products of two independent processes of infection and amplification following infection using hurdle generalized linear models (GLM). The hurdle GLMs were composed of a zero-truncated negative binomial GLM conditioned on a binomial GLM and fitted using the *R* package *glmmTMB* (54). Best-fitting models were selected by backward elimination of terms giving *P*  less than 0.05 starting from hurdle models with fixed effects of strain, heat treatment, and their interaction fitted in both binomial and negative binomial components. All *P* values were calculated using likelihood ratio tests.
