# Supplementary material for: High Temperature Cycles Result in Maternal Transmission and Dengue Infection Differences Between Wolbachia Strains in Aedes aegypti
Source: mBio. 2021 Nov 9;12(6):e00250-21. doi: 10.1128/mBio.00250-21 (PMC8576525; doi:10.1128/mBio.00250-21)

S1 Figure. Simulated larval, adult regime 1 and adult regime 2 (**A**) A representative 24-hour period of the simulated tropical water-temperature cycle generated from data from water drums known to act as *Ae. aegypti* larvae breeding sites in Trinidad (38). Data was collected using a water-proof temperature probe placed in a volume of water equal to that of the larval pans, and left in a dynamic temperature incubator. 24-hour period for regime 1 (**B**) and 2 (**C)** cycles for adult temperatures generated from data collected in urban Kuala Lumpur. Readings are from a temperature probe placed in a dynamic temperature incubator running the replica cycle.

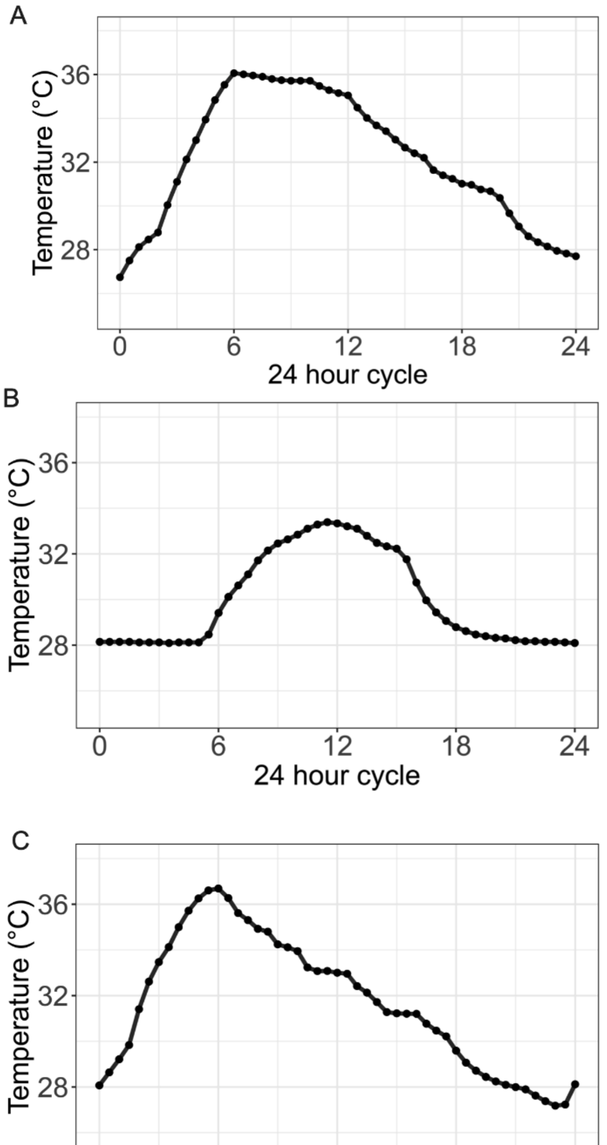

Supplement: FIG S1 [file mbio.00250-21-sf001.pdf]
