# Supplementary material for: High Temperature Cycles Result in Maternal Transmission and Dengue Infection Differences Between Wolbachia Strains in Aedes aegypti
Source: mBio. 2021 Nov 9;12(6):e00250-21. doi: 10.1128/mBio.00250-21 (PMC8576525; doi:10.1128/mBio.00250-21)

S2 Figure. Fluorescent *in situ* hybridization. Visualization of distributions and density reductions of *Wolbachia* (green) in ovaries of 5-days old females from *w*Mel, *w*AlbB and wild-type *Ae. aegypti* females from control and heat-treated groups. Blue stain is DAPI.

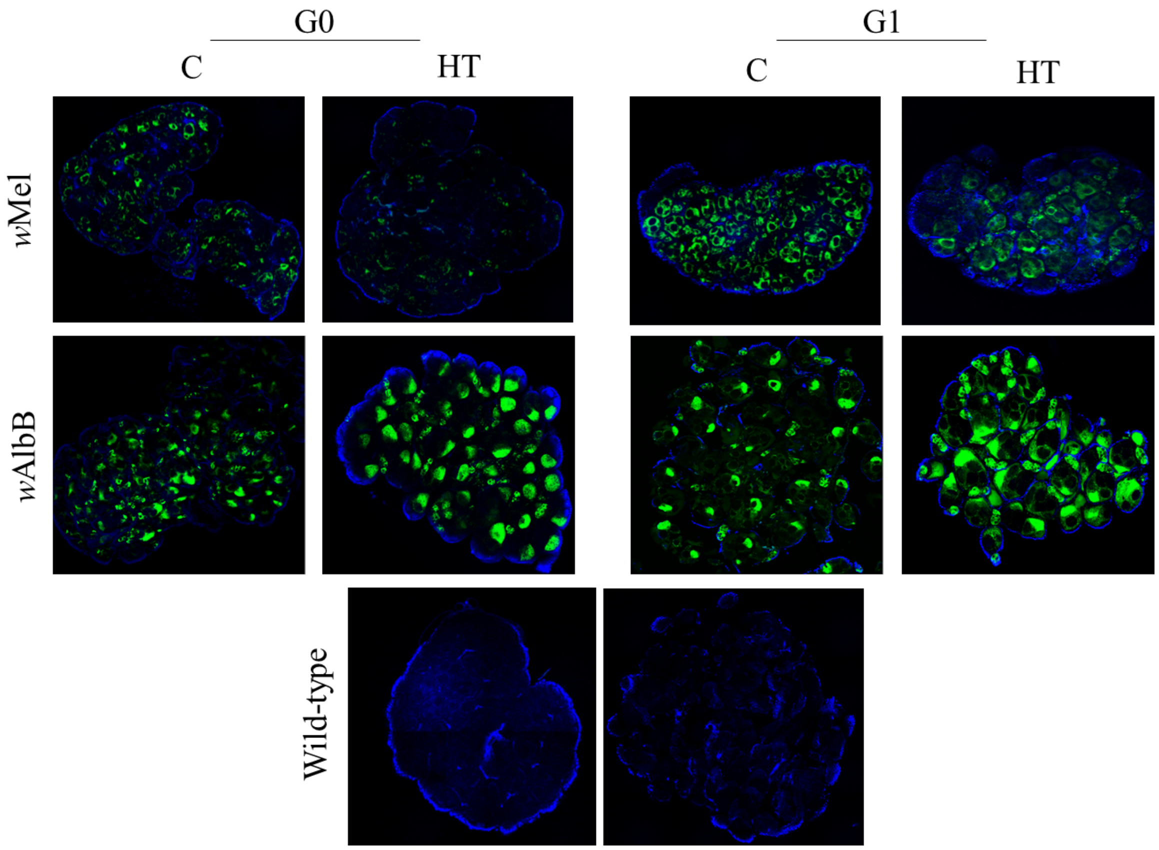

Supplement: FIG S2 [file mbio.00250-21-sf002.pdf]
