# Supplementary material for: High Temperature Cycles Result in Maternal Transmission and Dengue Infection Differences Between Wolbachia Strains in Aedes aegypti
Source: mBio. 2021 Nov 9;12(6):e00250-21. doi: 10.1128/mBio.00250-21 (PMC8576525; doi:10.1128/mBio.00250-21)

S3 Figure: DENV challenge on heat-treated wild-type, *w*AlbB- and *w*Mel-carrying Ae. aegypti. Salivary glands from heat-treated females were assessed for virus dissemination by Fluorescent Focus Assay (FFA). Dots represent the number of foci/ml per single mosquito (A). Dissemination rates from the same experiments are represented in panel B. Proportions on each bar indicate the infection rates (N positive/N total). Statistical analysis was performed using Mann-Whitney test and Fisher's exact test. '***' 0.001 '**' 0.01 '*' 0.05 'ns' not significant**.**

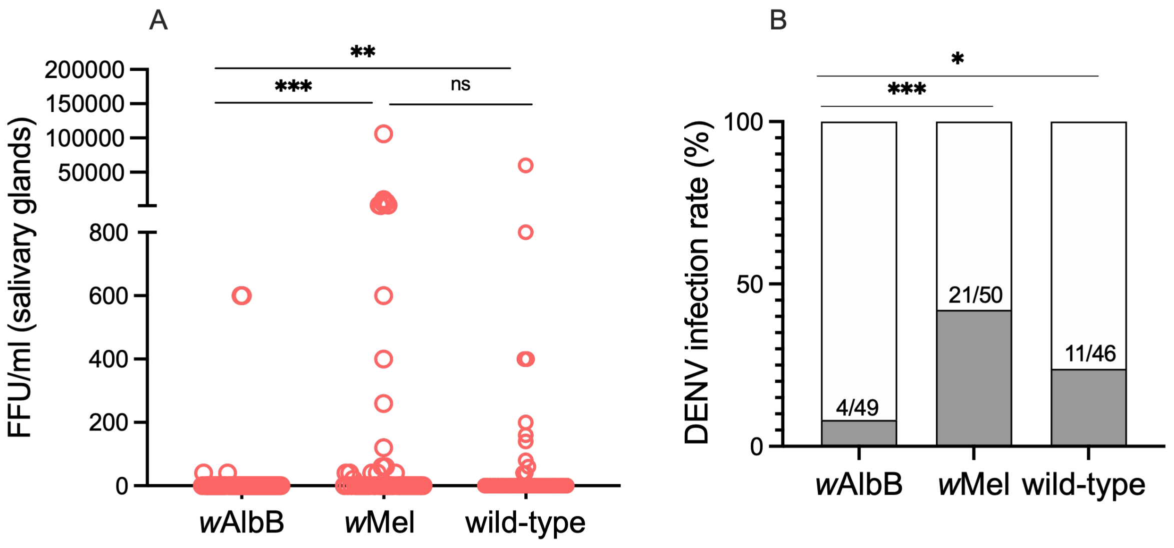

Supplement: FIG S3 [file mbio.00250-21-sf003.pdf]
