# Supplementary material for: High Temperature Cycles Result in Maternal Transmission and Dengue Infection Differences Between Wolbachia Strains in Aedes aegypti
Source: mBio. 2021 Nov 9;12(6):e00250-21. doi: 10.1128/mBio.00250-21 (PMC8576525; doi:10.1128/mBio.00250-21)
Supplement: TABLE S1 [file mbio.00250-21-st001.pdf]

S1 Table : List of sequences of oligonucleotides and probes**.**

**Primer name**	**5'-3' Sequence**
*w*AlbB-F **(7)**	GCAATACCTATGCCGTTTA
*w*AlbB-R **(7)**	GACGAAGGGGATAGGTTAATATC
*w*Mel-F **(7)**	TATTGAGCCTTCCTCGTACC
*w*Mel-R(29)	TAGCATGCCGTTTTTCTGTA
qHTH-F **(48)**	TGGTCCTATATTGGCGAGCTA
qHTH-R **(48)**	TCGTTTTTGCAAGAAGGTCA
qWSP-F (5)	ATCTTTTATAGCTGGTGGTGGT
qWSP-R **(48)**	GGAGTGATAGGCATATCTTCAAT
wsp probe W2**(5)**	CTTCTGTGAGTACCGTCATTATC-(Alexa Fluor 488)
wsp probe W3 **(5)**	AACCGACCCTATCCCTTCGAATA-(Alexa Fluor 488)
